# Supplementary material for: Blazed oblique plane microscopy reveals scale-invariant inference of brain-wide population activity
Source: Nat Commun. 2023 Dec 4;14:8019. doi: 10.1038/s41467-023-43741-x (PMC10695970; doi:10.1038/s41467-023-43741-x)
Supplement: Supplementary file 1 — Supplementary Information [file 41467_2023_43741_MOESM1_ESM.pdf]

Supplementary Information

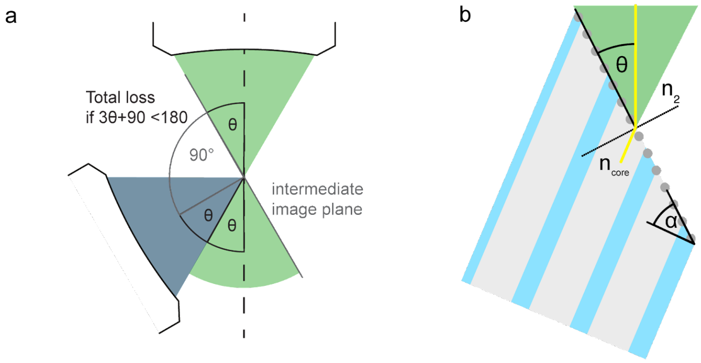

Supplementary Figure 1 | Re-imaging geometry a) Illustration of critical acceptance angle (θ=30°, NA=0.5 in air) at which conventional OPM fails b) One free parameter of the face plate design is the face plate angle, which can be chosen freely as the only requirement is that both surfaces are coplanar with the image surfaces of Obj_2 and Obj_3. However, coupling efficiency can be maximized by requiring the principal ray of the incoming cone to be refracted parallel to the fiber axes. This is achieved for $\alpha = arcsin\left(\frac{n_2}{n_{core}} \cdot cos(\theta)\right)$.

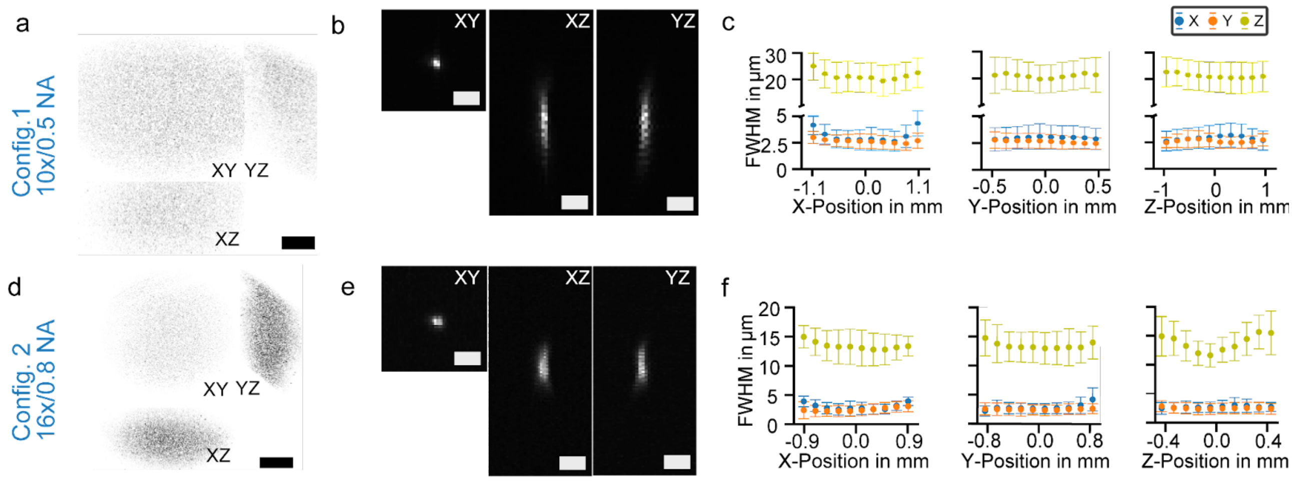

Supplementary Figure 2 | Resolution of the optical system a) Orthoprojections (maximum intensity projections, MIP) of a volume of fluorescent beads (d=1 µm, FOV 2.4 × 2.1 × 1 mm^3) imaged with the 10x configuration, b) orthographic MIPs of a single bead , c) mean and standard deviation of the measured full width at half maximum (FWHM, mean 2.7 ± 0.8 × 3.1 ± 1.1 × 21.1 ± 5.5 $µm^3$, n=17759) of beads across the FOV for all three axes show a relatively constant resolution. d) This procedure was repeated for the 16x configuration across a FOV of 2.1 × 1.7 × 0.8 mm^3. e) The axial extent with the increased NA is smaller and the sectioning capability is improved, f) which leads to an improved axial FWHM of 13.2 ± 2.8 µm (n=17684), but increased axial variability. Scale bars: a, d) 500 µm, b, e) 10 µm.

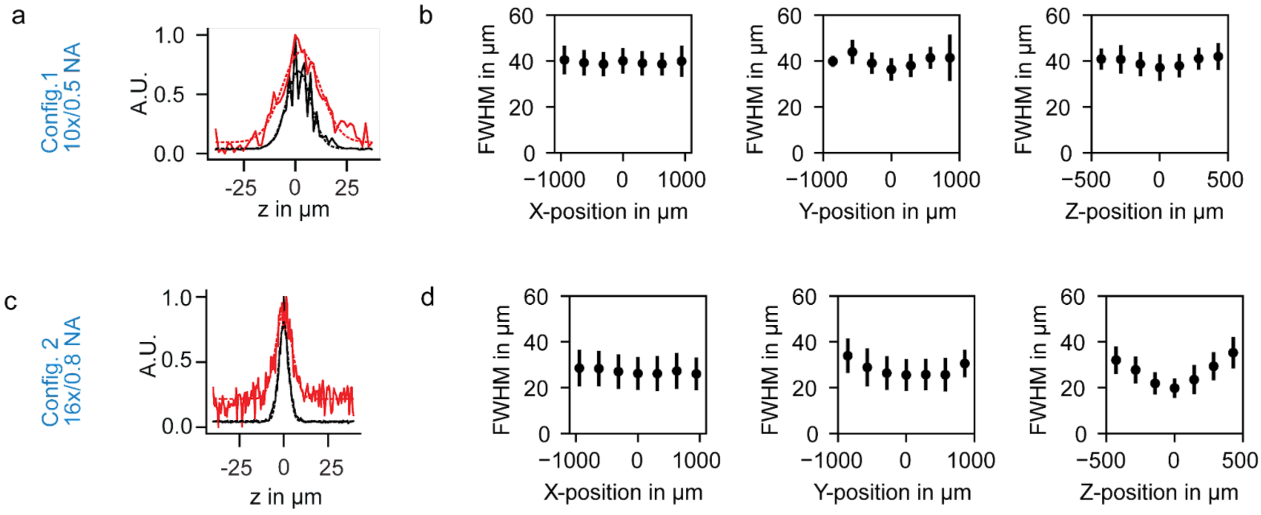

Supplementary Figure 3 | Quantification of the sectioning performance of both configurations a), c) Line plots of the axial intensity profile (black), the axial profile of the integrated intensity per plane (red, axial sectioning capability) and its Gaussian fit (dotted red) of a single bead for both configurations b),d) the FWHM of the axial section capability across the FOV (mean and standard deviation, b) n=514 , d) n = 2872).

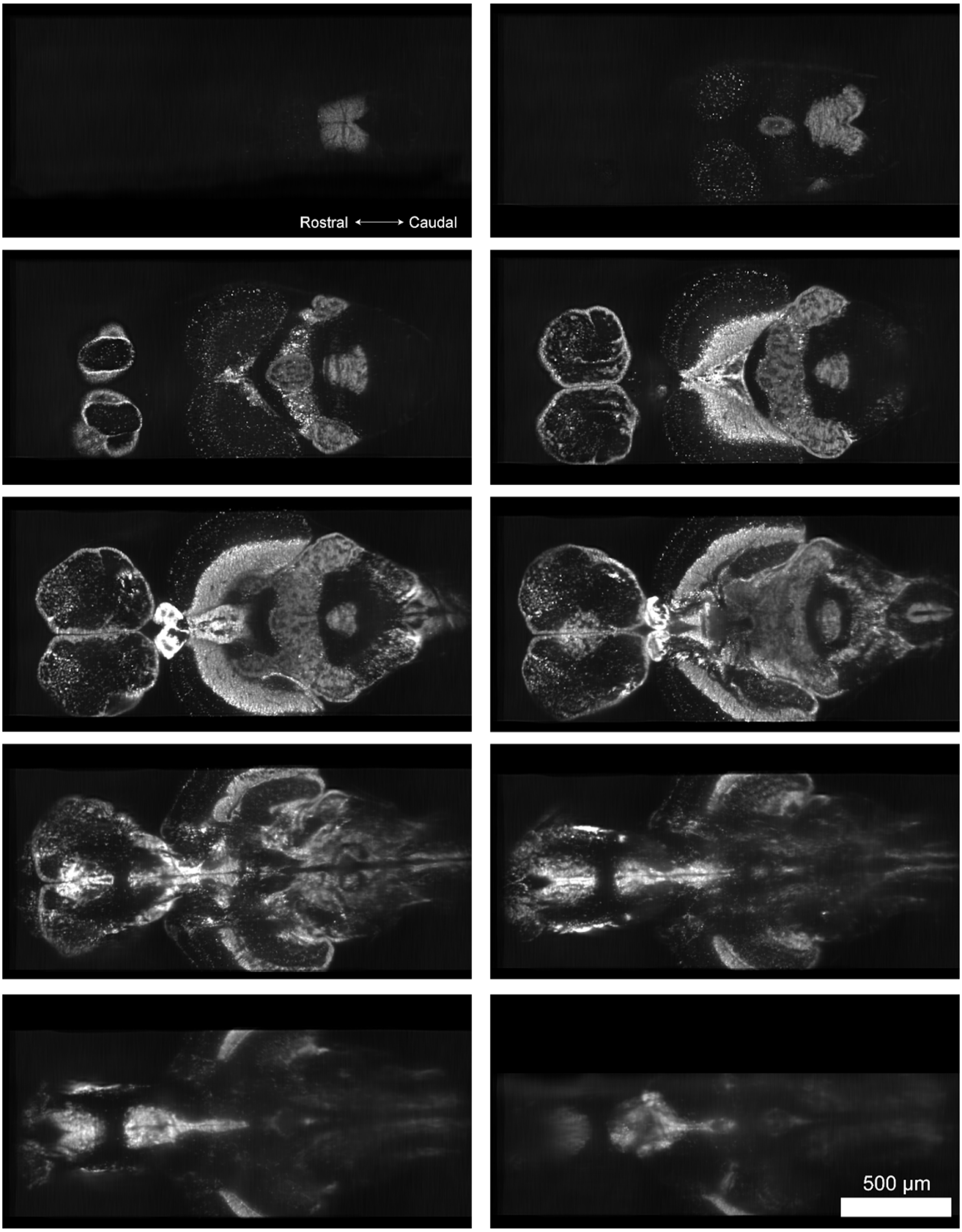

Supplementary Figure 4 | Slices of a single volume at different depths. Horizontal sections through a single representative imaging volume of the brain of DC from top left to bottom right in 60 µm steps – see Supplementary Movie 2 and 3.

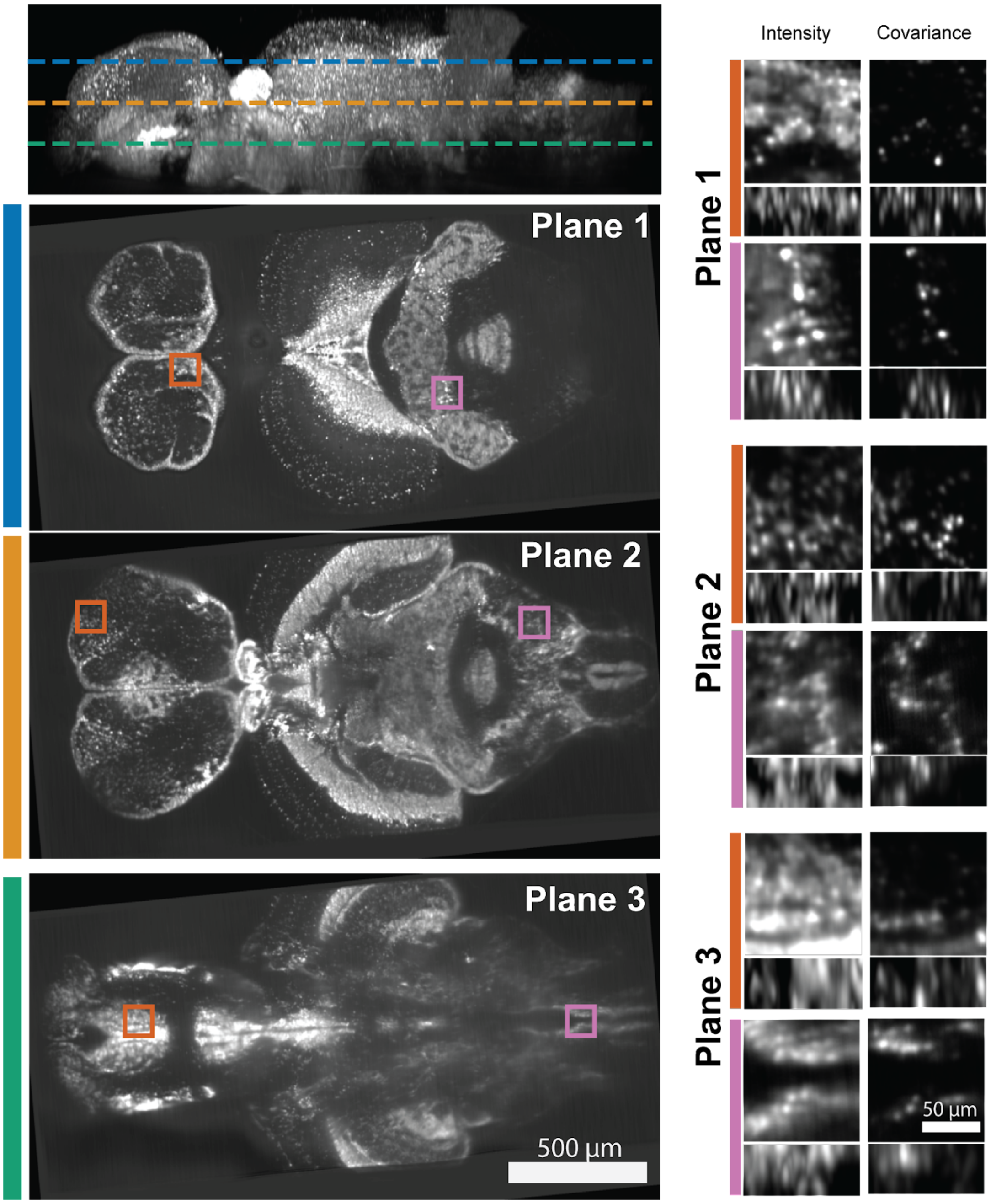

Supplementary Figure 5 | Anatomical resolution across depth: xy-sections of three planes situated at 280 µm, 430 µm and 570 µm from top of the imaging volume. For every xy-section we highlight two regions of interest by showing their intensity image (temporal average) and an orthogonal maximum intensity projection of the surrounding volume. This is displayed alongside the local covariance image we use for segmentation. This illustrates that the resolution of single cell nuclear can be found across many different brain regions across the imaging volume.

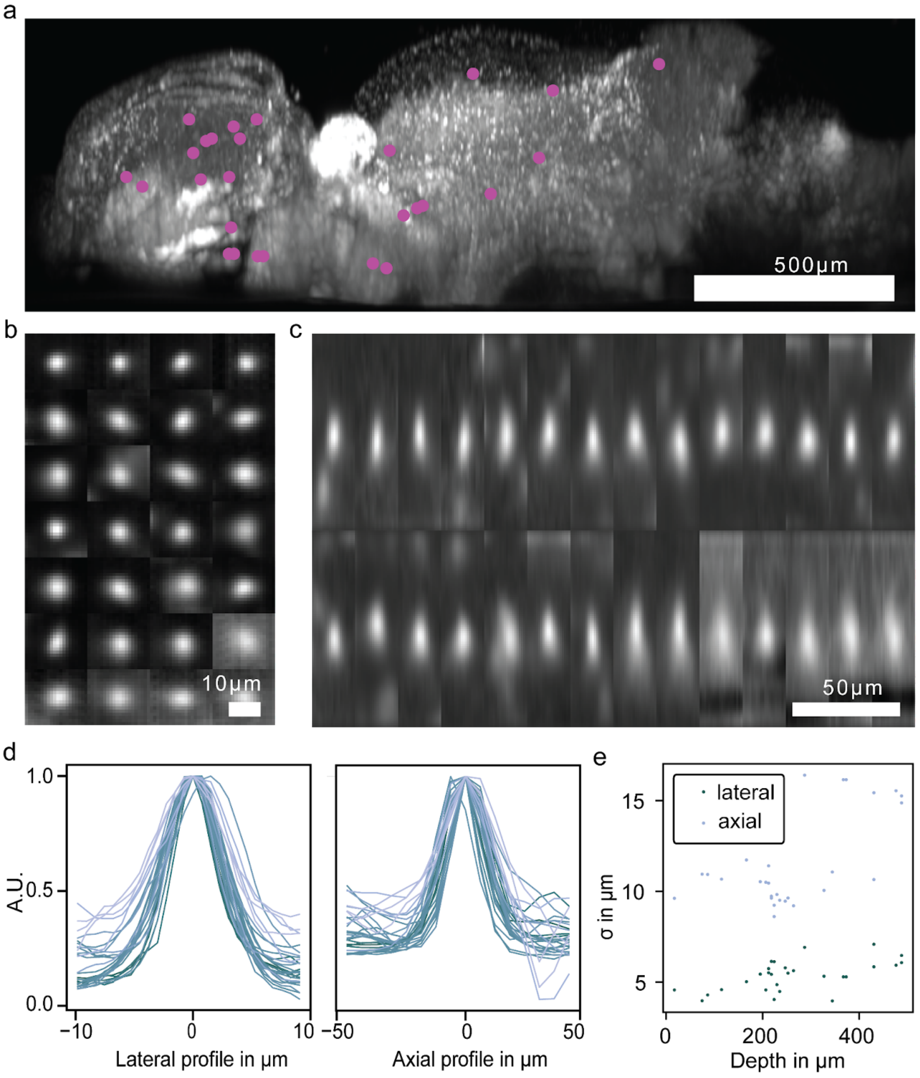

Supplementary Figure 6 | Quantification of effective cellular resolution as a function of depth. a) We identified isolated cell nuclei at various depths. b),c) Lateral (xy) as well as axial (xz) maximum intensity projections of these single cell nuclei. d) Measured lateral and axial intensity profiles of these nuclei color-coded by depth. e) Relationship between axial and lateral resolution (standard deviation of Gaussian fit) and cell depth.

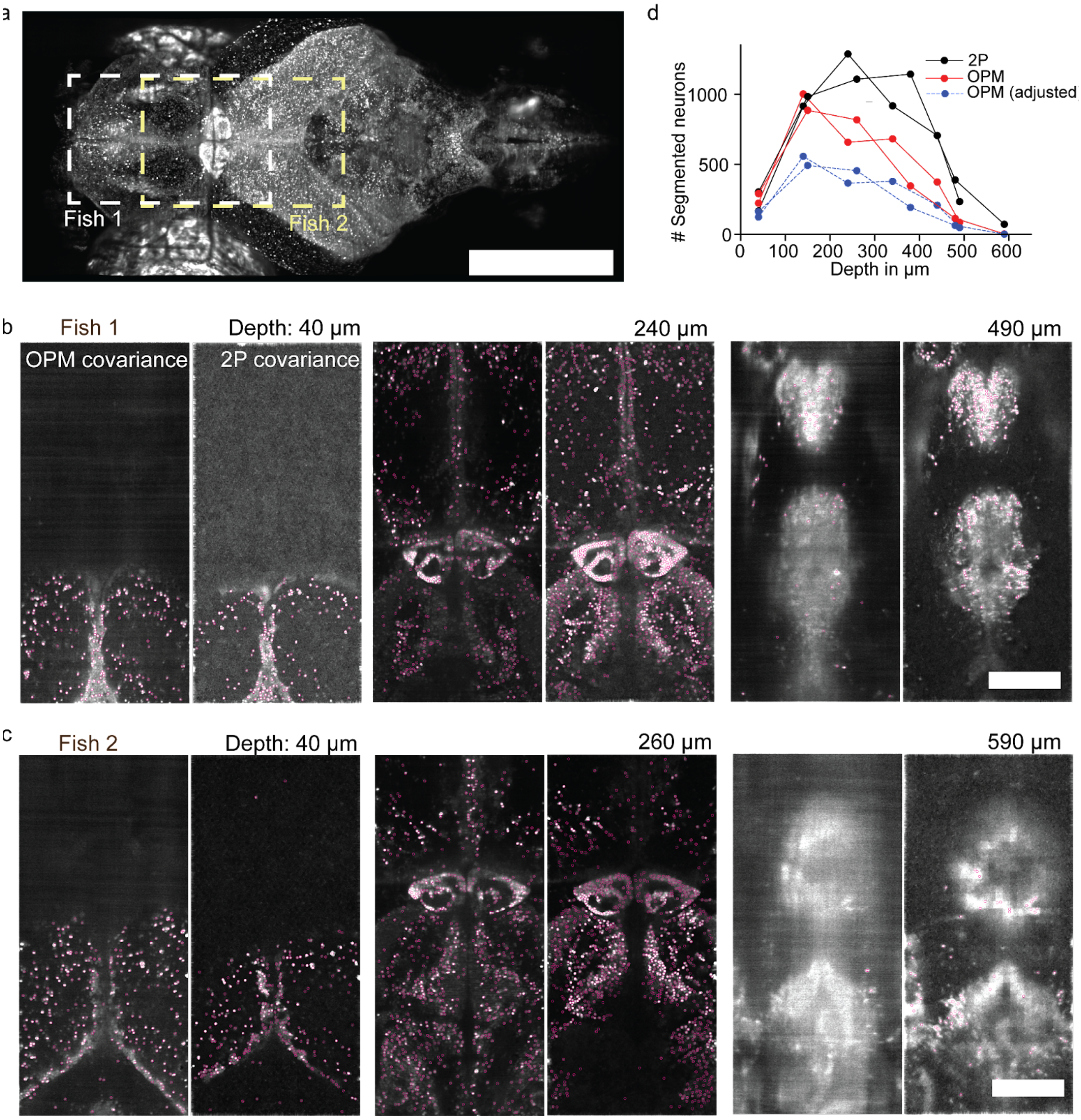

Supplementary Figure 7 | a) We performed blazed OPM followed by 2P microscopy in two fish. After imaging under the OPM for 600 s, we transferred the fish to a separate two-photon microscope and imaged one region per fish (dashed rectangle) at different depths (recording duration per plane: 600 s , 1 fps, 1024 x 480 pixel, 452.6 µm x 212.2 µm, 920 nm laser power: 6.3 - 22 mW). The size of the recording area was selected to match the frame rate of the OPM. scale bar: 500 µm. We manually determined and registered the corresponding planes of the OPM and the 2p recordings in both fish (top, middle and lowest plane are shown in b) and c), depth is given with respect to the top of the midbrain). d) We then segmented cells based on local covariance (see Methods). To account for the higher cell count in the OPM due to lower axial resolution we also plot a corrected cell count that is scaled by the approximate ratio of OPM and 2PM cell-PSFs (~1.8x); scale bar: 150 µm.

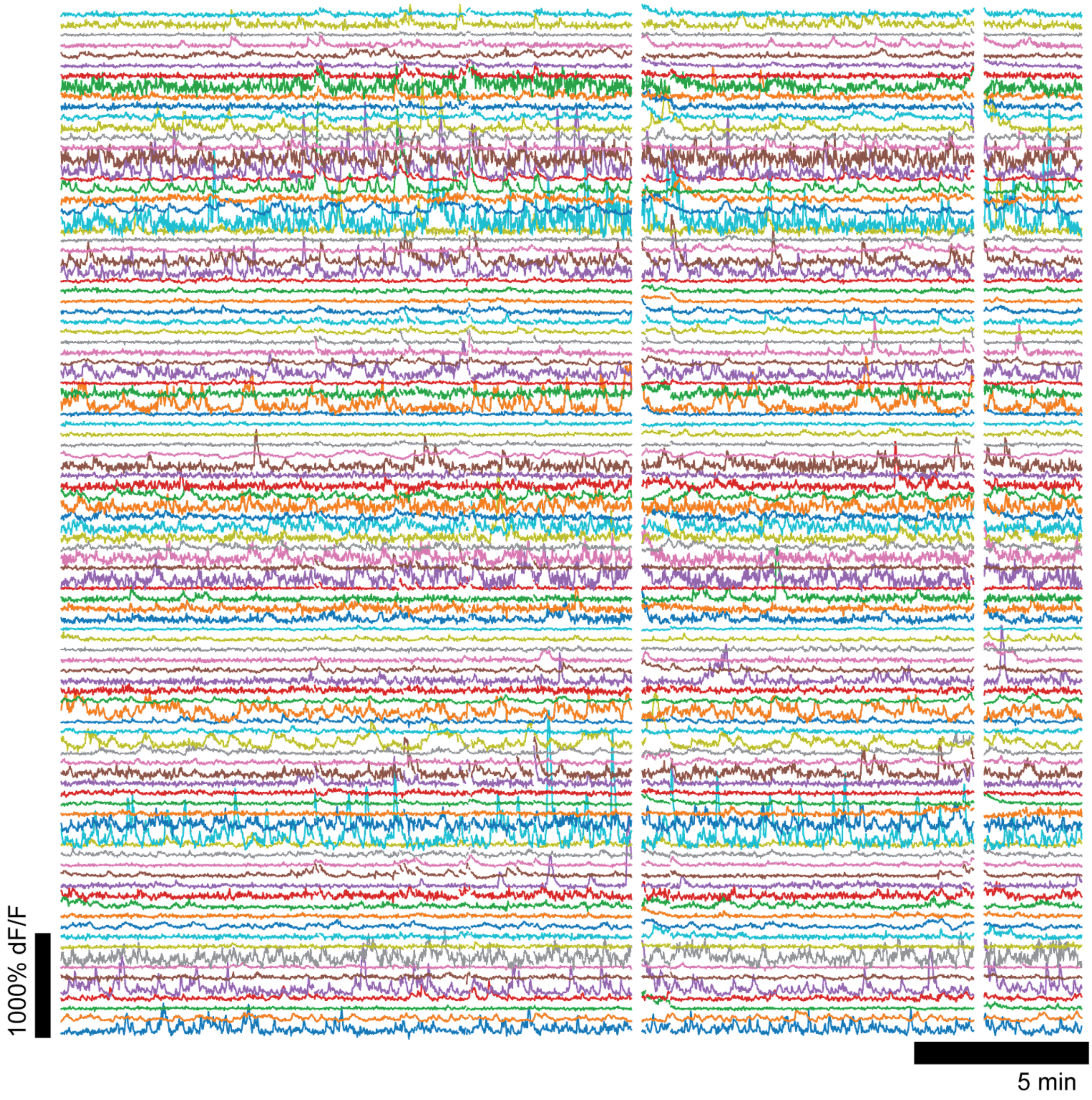

Supplementary Figure 8 | 100 randomly selected (by a computer) exemplary temporal traces. Blank spaces correspond to frames affected by motion artefacts.

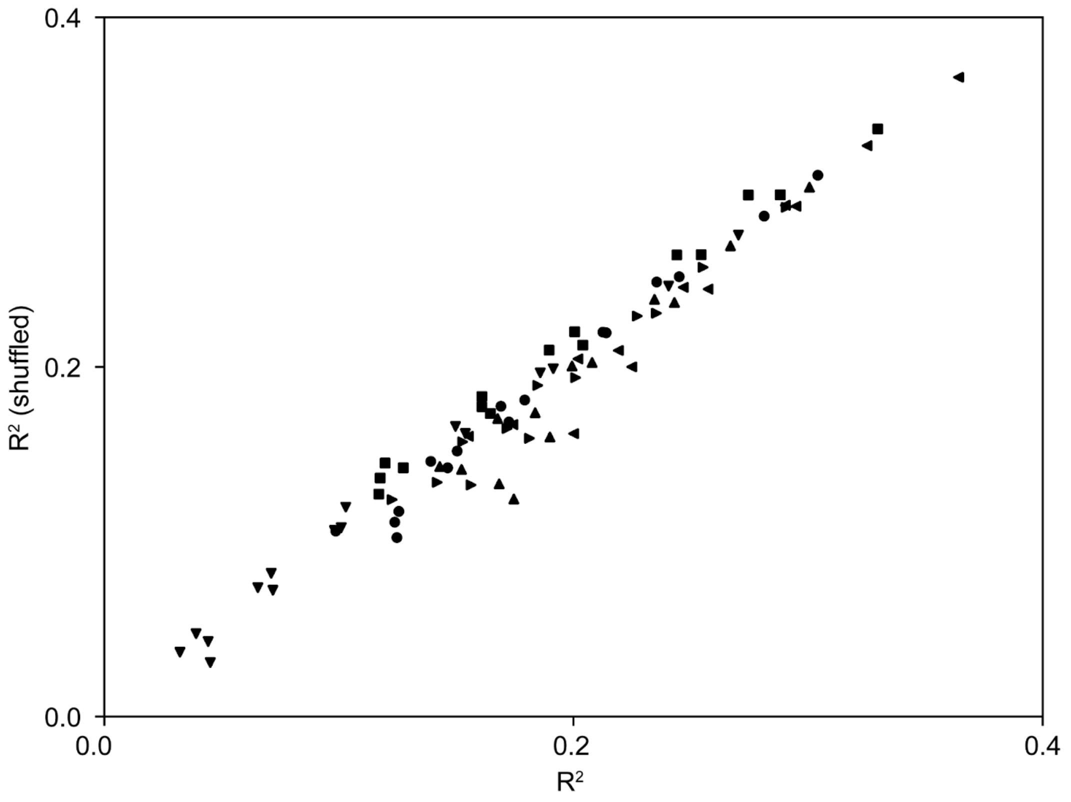

Supplementary Figure 9 | Obtained R^2 values across animals and different voxelizations (25 µm and larger, as in Figure 4d) for before and after spatial shuffling of cells. The ratio between R^2 of shuffled and non-shuffled data is 1.0 ± 0.1 (mean ± std, n = 45).

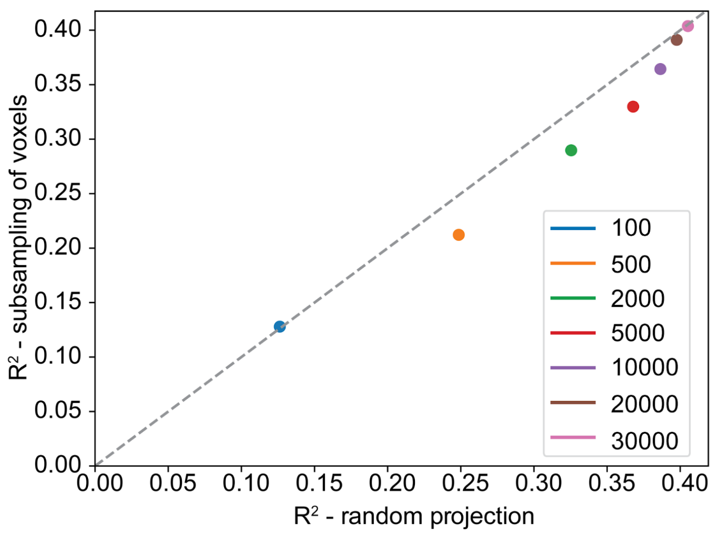

Supplementary Figure 10 | We compared the maximum R^2 achieved for a given number of predictors with the R^2 value reached with the same number of random projections of the brain-wide activity (excluding the prediction targets).

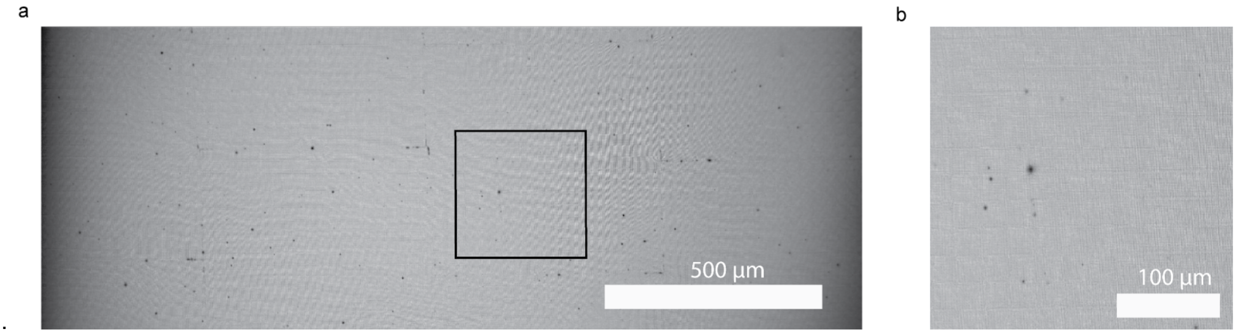

Supplementary Figure 11 | a) Camera image of the face plate surface (when homogeneously illuminating the input face), used as a correction pattern by which each camera frame was divided. b) Zoom in.

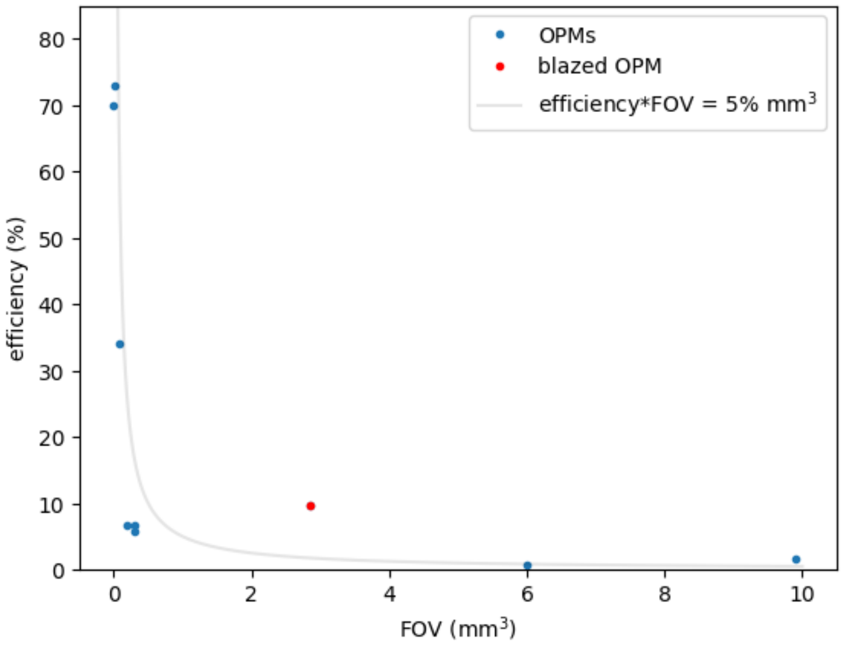

Supplementary Figure 12 | Plot of efficiency against FOV volume (data from Supplementary Table 1).

Method	eSPIM[1]	AMS-AGY[2]	SCAPE 2.0[3]	SoPi[4]	dOPM[5]	DaXi[12]	DOPM[6]	MesOPM[7]	Meso-OPM[8]	Blazed OPM
**Primary objective**	Water<br>60x<br>1.27 NA	Silicon<br>100x<br>1.35 NA	Water<br>20x<br>1.0 NA	Water<br>20x<br>1.0 NA	Water<br>40x<br>1.15 NA	Water<br>20x<br>1.0 NA	Air<br>4x<br>0.28 NA	Water<br>10X<br>0.5 NA	Air<br>10x<br>0.3 NA	Water<br>16x<br>0.8 NA
**Lateral resolution**	0.3 μm	0.3 μm	2.5 μm	1.3 μm	0.6 μm	0.5 μm x<br>0.4 μm	2.6 μm x<br>3.1 μm	1.5 μm x<br>2.8 μm	2.5 μm x<br>3 μm	2.8 μm ×<br>2.4 μm
**Axial resolution**	0.4 μm	0.7 μm	3.5 μm	-	1.33 μm	1.8 μm	37.4 μm	5.3 μm	6 μm	13.2 μm
**Near-aberration -free refocusing**	Yes	Yes	Yes	Yes	Yes	Yes	Yes	Yes	No	Yes
**Field of view (x × z) (width × depth)**	70 μm ×<br>20 μm	220 μm ×<br>60 μm	1100 μm ×<br>385 μm	950 μm ×<br>400 μm	295 μm ×<br>209 μm	800 μm ×<br>300 μm	3300 μm ×<br>1000 μm	1560 μm ×<br>240 μm	3300 μm ×<br>330 μm	2100 μm ×<br>800 μm
**Galvo scan range**	100 μm	180 μm	700 μm	500 μm	-	300 μm	3000 μm	780 μm	5500 μm	1700 μm
**Volume (mm^3)**	0.14*10^{-3}	2.4*10^{-3}	0.30	0.19	-	0.072	9.9	0.29	6.0	2.86
**Ratio of volume to resolution**	3.90E+06	3.80E+07	1.30E+07	-	-	2.00E+08	3.60E+07	1.30E+07	1.33E+08	3.20E+07
**Collection efficiency (at Obj1, % 2π)**	70 %	73 %	34 %	34 %	50 %	33.9 %	4.0 %	13 %	4.6 %	20%
**Efficiency of refocusing step:**	~1	~1	20 %[9]	20 %[10]	16 %	~1	43 %	~44%[11]	16 %	48 %
**Effective Collection Efficiency (% 2π)**	70 %	73 %	6.7 %	6.7 %	8 %	34 %	1.7 %	5.8 %	0.74 %	9.7 %

Supplementary Table 1 | Comparison of OPM techniques (Also see table in supplementary material of Ref.[12])

Supplementary References:

[1] Yang, B. et al. Epi-illumination SPIM for volumetric imaging with high spatial-temporal resolution. Nat. Methods 16, 501–504 (2019).
[2] Sapoznik, E. et al. A versatile oblique plane microscope for large-scale and high-resolution imaging of subcellular dynamics. Elife 9, (2020)
[3] Voleti, V. et al. Real-time volumetric microscopy of in vivo dynamics and large-scale samples with SCAPE 2.0. Nat. Methods 16, 1054–1062 (2019).
[4] Kumar, M. & Kozorovitskiy, Y. Tilt-invariant scanned oblique plane illumination microscopy for large-scale volumetric imaging. Opt. Lett. 44, 1706–1709 (2019).
[5] Sparks, H. et al. Dual-view oblique plane microscopy (dOPM). Biomed. Opt. Express 11, 7204–7220 (2020).
[6] Hoffmann, M. & Judkewitz, B. Diffractive oblique plane microscopy. Optica 6, 1166–1170 (2019). Singh, R. et al. Oblique plane microscope for mesoscopic imaging of freely moving organisms with cellular resolution. Opt. Express 31, 2292–2301 (2023).
[7] Singh, R. et al. Oblique plane microscope for mesoscopic imaging of freely moving organisms with cellular resolution. Opt. Express 31, 2292–2301 (2023).
[8] Shao, W. et al. Mesoscopic oblique plane microscopy with a diffractive light-sheet for large-scale 4D cellular resolution imaging. Optica 9, 1374 (2022).
[9] Calculated as light cone overlap between 0.75 NA Obj2 and 0.45 NA Obj3
[10] Calculated as light cone overlap between 0.75 NA Obj2 and 0.45 NA Obj3
[11] Calculated as light cone overlap between 0.45 NA Obj2 and 0.45 NA Obj3 with 25 deg sheet angle
[12] Yang, B. et al. DaXi-high-resolution, large imaging volume and multi-view single-objective light-sheet microscopy. Nat. Methods 19, 461–469 (2022).
