# Supplementary material for: Blazed oblique plane microscopy reveals scale-invariant inference of brain-wide population activity
Source: Nat Commun. 2023 Dec 4;14:8019. doi: 10.1038/s41467-023-43741-x (PMC10695970; doi:10.1038/s41467-023-43741-x)
Supplement: Supplementary file 3 — Description of Additional Supplementary Files [file 41467_2023_43741_MOESM3_ESM.pdf]

Title: Supplementary Movie 1:
Description: brain-wide imaging of a male Danionella cerebrum. Maximum intensity projection. Original recording was at 1Hz.

Title: Supplementary Movie 2:
Description: Z-stack video of a male Danionella cerebrum brain from dorsal to ventral. The mean intensity volume of the recording is followed by the temporal covariance map, which we use to segment cell nuclei (magenta circles).

Title: Supplementary Movie 3:
Description: Zoomed in version of a male Danionella cerebrum brain from dorsal to ventral. The mean intensity volume (green) of the recording is overlayed with the temporal covariance map (magenta), which we use to segment cell nuclei (yellow circles).
